# Health literacy: relationship with the perception of family functioning

**DOI:** 10.1515/med-2026-1432

**Published:** 2026-05-28

**Authors:** Carla Alexandra Silva Pombo Soares, Juliana Sofia Ortiga Nogueira, Nadirlene Pereira Gomes, Amâncio António de Sousa Carvalho

**Affiliations:** Nursing at Hospital da Luz, Avenida Carvalho Araújo , Vila Real, Portugal; Local Health Unit of Trás-os-Montes and Alto Douro, Douro Community Care Unit, Rua Dr. José de Sousa Pereira, Peso da Régua, Portugal; Federal University of Bahia, Nursing School, Salvador, Bahia, Brazil; Coordinating Professor of the Department of the School of Health, University of Trás-os-Montes e Alto Douro, Vila Real, Portugal; External integrated member of Research Centre on Child Studies, University of Minho, Braga, Portugal

**Keywords:** health literacy, family relationships, Family Health, family nursing

## Abstract

**Objectives:**

Health Literacy (HL) is influenced by health systems, education, the media, family, work environment, community and health policy decisions. The aim of the study is to analyze the relationship between the level of HL and the perception of family functioning (FF) on the selected sample.

**Methods:**

This is a descriptive-correlational, cross-sectional study with a quantitative approach, based on a sample comprised of 364 users of a Family Health Unit, in the north of Portugal. Data was obtained through the application of an online questionnaire, and analyzed using SPSS software, using descriptive and inferential statistics.

**Results:**

The largest group of users had a HL level classified as sufficient or problematic, both at 35.7 %. The majority (76.4 %) considered themselves a highly functional family. The statistical mean of the general HL Index differed between users with different perception of FF (ANOVA: p=0.031), with users who perceived their family as highly functional obtaining the highest score.

**Conclusions:**

We found that HL is related to FF, and families that perceive themselves as highly functional families have a higher level of HL, reinforcing the relevance of interventions aimed at promoting family functioning.

## Introduction

Health literacy (HL), although a relatively recent concept, has acquired increasing relevance in the fields of public health and health care due to its impact on population health outcomes [1]. Since the emergence of its first definitions in the late 20th century, HL has evolved from a concept focused on individual task performance to a broader construct encompassing both personal and social dimensions, enabling individuals to make informed decisions in their daily lives and assume responsibility for those decisions [2].

In this context, HL is defined as the ability to obtain, process, understand, evaluate, and apply health information, enabling the appropriate use of health services and informed decision-making regarding care, disease prevention, and health promotion [3], 4]. Its dynamic nature requires individuals to continuously update their knowledge, integrate new evidence, and adapt to changes in health systems and societal contexts [5].

HL assessment plays a critical role in guiding interventions and supporting the development of health policies tailored to population needs [6]. Measuring HL enables the identification of inequalities in accessing, understanding, and using health information, while strengthening the knowledge and evidence base required to inform the design and implementation of effective public health strategies across the life course [3], 7].

In recent years, several studies have been conducted in Portugal to assess the level of HL in different contexts and with different populations. Some of these studies are cited in the discussion of the results of the empirical study in question. In 2022, the Directorate-General for Health carried out the HLS19 study in Portugal, using a reduced version of the European Health Literacy Survey (HLS-EU-PT). The results revealed that 7 out of 10 people in the country have high levels of HL, while 30 % of the population has problematic or inadequate levels [7].

Family functioning (FF) is another key determinant of health, influenced by social, cultural, and economic transformations that shape family roles, processes, and resources [8], and reflects patterns of communication, power distribution, adaptability, and interaction among family members, which directly affect decision-making processes, coping strategies, and overall health regulation [9]. Families continuously adapt to normative and non-normative transitions across the life course, and their dynamics may either promote or hinder health and well-being [10], 11].

Assessment of FF is essential to identify dysfunctions in family dynamics and guide effective therapeutic interventions. Understanding these interactions not only helps prevent mental and physical health problems but also informs public policies and family support programs. This assessment is crucial for healthcare professionals to provide more holistic care, promoting healthy family environments. Several instruments have been developed to evaluate key dimensions of FF, including communication, cohesion, adaptability, and support, enabling the identification of interaction patterns and family needs [12], [13], [14].

In Portugal, the inclusion of the family as the focus of nursing care and the consolidation of the role of the family nurse in Primary Health Care (PHC) follow the conceptual framework and international guidelines of the World Health Organization (WHO), being reinforced by Decree-Law Nr. 118/2014 [15], which emphasizes the relevance of nursing in health promotion and family-centered care.

As HL is a dynamic process that requires continuous updating and critical capacity, the family, as a support and socialization system for health, assumes a central role in its promotion. In this sense, the quality of family interactions can positively or negatively influence HL, therefore studying the relationship between HL and FF allows identifying key factors for more effective interventions [16].

The present study is justified by the growing relevance of HL in public health, as well as by the importance of FF as a facilitating context in the development of these competencies. Within this framework, the study was guided by the hypothesis that a relationship exists between the level of HL and the perception of FF. The underlying research question was: what is the relationship between the level of HL and the perception of FF? Accordingly, the present study aims to analyze the relationship between the level of HL and the perception of FF in the studied sample.

## Methods

The present research is a descriptive-correlational, cross-sectional study with a quantitative approach. The research took place in a Family Health Unit (FHU) located in the North of Portugal, relating the level of HL and the perception of FF, establishing relationships between variables, but did not seek to establish a cause-effect relationship [17].

### Population and sample

FHU are key structures in the provision of primary healthcare with the mission of delivering personalized medical care to the population of a defined geographical area, ensuring accessibility, comprehensiveness, quality, and continuity [18]. Users are all individuals and families registered with the FHU. This service is considered public, having been created in 1979, with the objective of guaranteeing all citizens the fundamental right to health protection, enshrined in the Constitution of the Portuguese Republic [19].

The population comprises approximately 4,449 users and was defined based on the following inclusion criteria: i) Users registered at the FHU under study; ii) Users aged 24 to 44 at the FHU under study.

The 24–44 age group was selected because, according to national data, it represents a population with high levels of digital competence and widespread internet use. Within this age range, digital communication practices such as email use, are widely disseminated, reflecting the integration of digital technologies into academic, professional, and social spheres [20], 21].

As it was not possible to study the entire population, a representative sample was defined taking into account the following exclusion criteria: i) Users who did not have an email available in the FHU administrative process under study; ii) Users who changed of FHU during the data collection period; iii) Users who did not answer at least 14 items on the HLS-EU-PQ16 scale.

The minimum sample size calculated was 367 users, aged between 24 and 44 years old, enrolled in the FHU under study. From a population of 4,449 users, we apply the Araldi formula n=[(Nxn_0_)/(N+n_0_)], using a sampling error of 5 % and a 95 % confidence interval. The n is the minimum sample size, N is the population size, and n_0_ corresponds to the first approximation to the sample size, calculated from formula 1/(E_0_), where E_0_=0.05. The minimum sample size is calculated as n=(4,449 × 400)/(4,449+400)=367 users [22]. In total, 377 users responded to the questionnaire, and 10 additional questionnaires to maintain the minimum sample size after applying the exclusion criteria. However, after applying the exclusion criteria, the final sample consisted of 364 participants, of which 13 users were excluded for not having responded to at least 14 items on the HLS-EU-PTQ16 scale.

The sampling adopted was non-probabilistic, convenience sampling, which means that the various individuals in the population did not have the same probability of being included in the sample [17].

### Data collection instrument

In the present study, primary data collection was carried out using a questionnaire organized into three sections. The first section of the questionnaire consisted of questions aimed at the sociodemographic characterization of users. The second section was part of the European Health Literacy Survey scale – reduced version (HLS-EU-PT-Q16), validated for the Portuguese population by [23] whose objective is to assess the level of HL, based on the individual’s self-perception. The third section consisted of questions related to the perception of FF, measured through the application of the Family Apgar Scale, constructed by Smilkstein [14] and validated by Smilkstein et al. [24] and adapted to the Portuguese version by Agostinho and Rebelo [25].

The Smilkstein Family Apgar Scale, in the adapted Portuguese version, is freely accessible for use in non-commercial contexts. Regarding the HL European Health Literacy Survey Scale – reduced version (HLS-EU-PT-Q16), due consent was requested and obtained for its use in the present study.

#### European health literacy survey (short version) HLS-EU-PT-Q16

The HLS-EU-PT-Q16 scale consists of 16 items developed based on the Integrated Conceptual Model of HL, proposed by Sørensen et al. [26]. The scale covers three health domains: Health Care (HC) – 7 items, Disease Prevention (DP) – 5 items, and Health Promotion (HP) – 4 items. These domains are assessed through four levels of information processing: access (items 1, 2, 8, 13), understand (items 3, 4, 9, 10, 14, 15), evaluate (items 5, 11, 16) and use (items 6, 7, 12). These levels are directly related to the ability to make informed decisions to maintain health, prevent disease, and seek appropriate treatment when necessary.

For the HL assessment to be considered valid, the users must respond to at least 14 of the 16 items on the scale, which constitutes an exclusion criterion. Respondents must indicate the degree of difficulty perceived in carrying out tasks related to the three assessed domains. The answers are classified into two categories: “difficult” and “very difficult”, corresponding to a 0 score; and “easy” and “very easy”, corresponding to a one score. The sum of the scores assigned to the items results in the individual HL level, with the following classification: 0 to 8 points inadequate HL level; 9 to 12 points problematic level and 13 to 16 points adequate level.

Additionally, for a more detailed analysis, the results were standardized by calculating a general HL index (G-HL16 index), converted to a scale from 0 to 50, where 0 represents the minimum level and 50 the maximum HL level. This index was calculated according to the formula: G-HL16 Index=[(X−1) × (50/3)], where X is the average of the values attributed to the responses to the items (from one to 4, as follows: 1=“very difficult”, 2=“difficult”, 3=“easy”, 4=“very easy”). Based on this index, HL was classified into four levels: inadequate (0–25), problematic (25.1–33), sufficient (33.1–42), excellent (42.1–50) [23], 27].

Cronbach’s alpha coefficient, used to assess the internal consistency of the HLS-EU-PT-Q16 scale, presented the following values: 0.783 in the HC domain, 0.724 in DP domain, 0.703 in HP domain and 0.890 in the total scale, indicating high overall reliability.

#### Smilkstein’s family APGAR scale

The Family APGAR Scale is based on the premise that family members can assess FF and express their degree of satisfaction with the fulfillment of the basic functions of this system. The acronym APGAR represents five fundamental components of FF, as defined by Smilkstein. The letter A for Adaptation refers to the family’s ability to use internal and external resources to deal with crisis situations, seeking to restore family balance. The letter P for Partnership refers to the sharing of responsibilities and joint decision-making among family members, promoting an environment of cooperation and co-responsibility. The letter G for Growth relates to the physical and emotional development and self-realization of family members, made possible by mutual support and shared guidance. The letter A for Affection translates to the existence of emotional bonds between family members, expressed through care, affection, and emotional closeness. The letter R for Resolve implies a commitment by family members to dedicate time to each other, offering physical and emotional support, as well as sharing resources and family space harmoniously [14].

The Family APGAR Scale consists of five closed-answer questions that assess the degree of satisfaction of individuals in relation to the dimensions of FF represented by the acronym APGAR. The answers are presented in three options: “almost always”, “sometimes” and “almost never”, which are assigned scores of 2, 1 and 0, respectively [14]. The total score on the scale is the result of the sum of the values attributed to the five questions and can vary between 0 and 10 points. Based on this total, it is possible to categorize FF as follows: severe family dysfunction: 0 to 3 points; Mild family dysfunction: 4 to 6 points; High family functionality: 7 to 10 points. This categorization follows the proposal of the Portuguese adaptation of the scale carried out by Agostinho and Rebelo [25].

### Data collection

The data collection source was comprised by users registered at the FHU under study who answered the questionnaire, ensuring they met the defined inclusion and exclusion criteria. The data was obtained electronically, using the questionnaire mentioned above, through the free online platform Google Forms^®^, which generates a link for users to access the data collection instrument and complete it. The questionnaire link was sent by email to users who had an email address in their medical records, with data collection taking place between January and February 2024.

### Ethical approval

The questionnaire was prepared with consideration toward the dignity and rights of the study participants. The answers do not allow for individual identification, as the aim was to only obtain the essential personal data for carrying out the study [17].

The data collection instrument did not request personally identifiable information, granting anonymity and confidentiality. The users were given the option to refuse participating in this study at any time, without any consequences.

The questionnaire begins with an introductory note containing the title of the study, its objective and the identification of the authors, as well as a statement that data collection would be anonymous and confidential and that the participant could stop completing it at any time. A mandatory question was designed: “Do you declare that you have read and understood the clarification provided in the previous section and agree to participate in this study?”, in which the respondent had to answer affirmatively (yes) to continue with the questionnaire, thus safeguarding the Informed, Free and Informed Consent for participation in research.

In the development of this study, the ethical principles inherent in the Declaration of Helsinki and the Oviedo Convention were respected. A request for authorization to carry out the study was made to the Health Unit Coordinator and to Group of Health Centers Marão Douro Norte, including the endorsement of the person responsible for Access to Information and the respective favorable opinions were obtained. A request for authorization was also made to the Ethics Committee of the North Regional Health Administration, and a favorable opinion was obtained (Opinion CE/2024/08 of 08-01-2024).

### Data processing and analysis procedure

In this study, the statistical analysis of the data aimed to answer the formulated research questions. The collected data was properly organized, coded, and entered in a database created using the Statistical Package for the Social Sciences (SPSS, version 29.0) software.

We did assess and process database error inputs and performed a descriptive and inferential analysis of the data. Regarding descriptive statistics, absolute and relative frequencies were calculated for all variables, and measures of central tendency and dispersion for scalar-level variables.

Regarding inferential statistics, the ANOVA and Kruskal-Wallis tests were used. The assumptions for the use of parametric tests were ensured, specifically, normality (Kolmogrov-Sminorv test) and homogeneity of variances (Levene test). When the variables did not meet the assumptions (did not present a normal distribution or dimensions smaller than 30), the nonparametric test was applied. In the case of the cross-tabulations between Health Literacy Index categories (HLI Cat) and the Family Functioning variable, this was this variable is ordinal by categories. To calculate the effect sizes for each analysis, we used the eta squared (η^2^), in the case of the ANOVA test, and the epsilon squared (e^2^), in the case of the Kruskal-Wallis test, reinforcing the interpretation of the practical relevance of the statistically significant results. The significance level considered was 5 % [28].

To assess whether the categories of FF, sex, age group, educational level, and marital status had a statistically significant effect on the response probabilities of the HLI cat, an ordinal regression with a “positive *log-log” link* function was used. The choice of this function was based on the analysis of the frequency distribution of the classes of the dependent variable. Although other link functions, such as the “***logit***”, were tested, the “***positive log-log***” function showed the best statistical significance. The model’s assumption of homogeneity was validated [28].

## Results

Of the total sample (n=364 users), the majority were female (69.0 %), in the 34–44 age group (62.4 %), had higher education qualifications (60.7 %) and were married or living in a common-law union (63.7 %) (Table 1).

**Table 1:** Sociodemographic characterization of the study sample (n=364).

Variables		Absolute frequency	Relative frequency
Gender	Male Female	113 251	31.0 69.0
Age group	≥24–33 years ≥34–44 years	137 227	37.6 62.4
Literary qualifications	Basic education Secondary school Higher education	23 120 221	6.3 33.0 60.7
Marital status	Single Married/common-law union Divorced/widowed	114 232 18	31.3 63.7 4.9

To assess HL levels, the HLS-EU-PT-Q16 scale was applied using the formula that uses the sum of the scores for each item. The majority of users (65.7 %) had adequate HL, 22.8 % had problematic HL and 11.5 % had inadequate HL.

The other results presented were obtained using the formula that used the sum of the scores of the 16 items, with responses ranging from one to 4. The percentages of the response types “Difficult and Very difficult”, and “Easy and Very easy” were associated in each of the items and it was found that the items that contributed most to a higher literacy level, in decreasing order, that obtained higher percentages in the response options “Easy” and “Very easy” were item 7 “Follow instructions from your doctor or pharmacist?” with 96.4 %, item 4 “Understand the instructions from your doctor or pharmacist about taking a prescribed medication?” with 96.1 %” and item 3 “Understand what your doctor tells you?” with 93.0 %. In turn, the items that contributed least to a higher level of literacy, in decreasing order, which obtained higher percentages in the response options “Very difficult” and “Difficult” were item 8 “Finding information to deal with mental health problems such as stress or depression?”, item 12 “Deciding how to protect yourself from illness based on information in the media?” and item 5 “Assess when you may need a second opinion from another doctor?”, which obtained a percentage of 37.9 , 29.1 and 26.9 %, respectively. Item 8 also presented the highest number of “don’t know” responses with 3.3 % (Table 2).

**Table 2:** Percentage of responses in each of the options to the 16 items of the HLS-EU-PT-Q16 (%).

Dimension	Item Processing levels	On a scale of very easy to very difficult how easy would you say it is:	1 Very difficult, %	2 Difficult, %	3 Easy, %	4 Very easy, %	5 Don’t know, %
Health care	I1 To access	Looking for information on treatments for conditions that concern you?	1.6	19.8	58.8	18.6	1.6
	I2 To access	Want to know more about where to get expert help when you’re sick?	1.6	19.0	58.5	20.3	0.5
	I3 To understand	Understand what your doctor tells you?	0.5	6.3	64.6	28.6	0.0
	I4 To understand	Understand your doctor or pharmacist’s instructions about taking a prescribed medication?	0.3	3.3	60.7	35.4	0.3
	I5 To assess	Assess when you might need a second opinion from another doctor?	1.6	25.3	54.7	17.3	1.9
	I6 To use	Use information your doctor gives you to make decisions about your illness?	0.8	14.0	65.1	18.1	1.9
	I7 To use	Follow your doctor or pharmacist’s instructions?	0.3	3.3	60.4	36.0	0.0
Disease prevention	I8 To access	Finding information to deal with mental health issues like stress or depression?	7.4	30.5	46.2	12.6	3.3
	I9 To understand	Understand health warnings regarding behaviors such as smoking, talking about physical activity and excessive alcohol consumption?	0.8	8.5	56.0	33.5	1.1
	I10 To understand	Understand why you need to do screenings?	0.5	6.6	56.6	35.7	0.5
	I11 To assess	Assess whether information in the media about health risks is reliable?	3.0	23.1	56.3	16.2	1.4
	I12 To use	Deciding how to protect yourself from the disease based on media information?	4.4	24.7	57.7	12.1	1.1
Health promotion	I13 To access	Want to learn more about activities that are good for your mental well-being?	1.4	17.3	59.1	21.4	0.8
	I14 To understand	Understanding health advice from family or friends?	2.7	23.1	55.8	17.3	1.1
	I15 To understand	Understand the information in the media about how to stay healthier?	1.1	16.8	60.4	21.2	0.5
	I16 To assess	Assess which daily behaviors are related to your health?	0.8	12.9	63.7	22.5	0.0

Caption: I, item.

It was found that the “Disease Prevention” dimension obtained the highest percentage of responses in the “Very difficult” and “Difficult” options with 37.9 % (item 8), at the “Access” processing level, followed by the “Health Care” dimension with 29.1 % (item 12) at the “Use” processing level and, lastly, the “Health Promotion” dimension with 25.8 % (item 14), at the “Understand” processing level.

In the HL indexes (HLI), the average of the highest score obtained is that of the “Understand” processing level with 35.68 points, followed by the average of the HC Dimension index with 34.59 points, which fell within the Sufficient HL level. The HLI that obtained the lowest averages, in ascending order, were that of the “Access” processing level with 30.95 points, followed by the DP Dimension index with 32.07 points, which fell into the “Problematic” level. It is worth highlighting the fact that the average of the DP Dimension Level and the “Access” processing level fell into the Problematic level (Table 3).

**Table 3:** Measures of central tendency and dispersion of the general index, dimensions and levels of information processing of the HL.

Variables	n	Mean	Median	Mode	SD	Min.	Max.
General HL index	364	33.41	33.33	33	7.68	4	50
HC dimension index	364	34.59	33.33	33	8.06	2	50
DP dimension index	364	32.07	33.33	33	9.03	3	50
HP dimension index	364	33.04	33.33	33	9.24	8	50
Processing level index access	364	30.95	33.33	33	9.67	0	50
Processing level index understand	364	35.68	33.33	33	8.10	3	50
Processing level index assess	364	33.02	33.33	33	9.41	0	50
Processing level index use	364	33.56	33.33	33	8.42	6	50

Caption: DP, disease prevention; HC, health care; HL, health literacy; HP, health promotion; Min., minimum; Max., maximum; n, total sample; SD, standard deviation.

Regarding the categorization of the General HL Indexes and the Dimensions of the sample, the highest percentages were, in all indexes, at the Sufficient HL level, with the highest percentage at this level being that of the HP Dimension, while the lowest is that of the General HL index (35.7 %), a percentage that in this index coincides with that of the Problematic level. If we associate the percentages of the Inadequate and Problematic levels of the General HL index, they total a percentage of 49.2 %, almost half of the sample. The HL level that obtained the highest percentage at the Excellent level was that of the HC Dimension (20.9 %), with the General HL index presenting the lowest percentage (Table 4).

**Table 4:** Categorization of health literacy, health care, disease prevention and health promotion indices of the sample (n=364).

Variables	Inadequate		Problematic		Sufficient		Excellent	
	0–25 points		25.1–33 points		33.1–42 points		42.1–50 points	
	n	%	n	%	n	%	n	%
General HL	49	13.5	130	35.7	130	35.7	55	15.1
HL health care	29	8.0	101	27.7	158	43.4	76	20.9
HL disease prevention	70	19.2	99	27.2	136	37.4	59	16.2
HL health promotion	74	20.3	55	15.1	176	48.4	59	16.2

Caption: HL, health literacy.

Regarding the perception of FF, as shown in Table 5, most users responded “Almost always” to all items evaluated. The items with the highest percentage of responses in this category were item 1 (Adaptation), with 74.7 %, and item 3 (Growth), with 65.7 %. On the other hand, the lowest percentages of “Almost always” responses were observed in items 5 (Decision), with 53.0 %, and 4 (Affection), with 61.3 %.

**Table 5:** Percentage obtained for each response option to the different items on the Smilkstein family apgar scale (APGAR).

APGAR	Variable	Almost never		Sometimes		Almost always	
		n	%	n	%	n	%
Adaptation	I1- I am satisfied with the help I receive from my family whenever something worries me	14	3.8	78	21.4	272	74.7
Partnership	I2- I am pleased with the way my family discusses matters of common interest and shares with me the solution to the problem	23	6.3	07	29.4	234	64.3
Growth	I3- I think my family agrees with my desire to embark on new activities or change my lifestyle	26	7.1	99	27.2	239	65.7
Affection	I4- I am pleased with the way my family expresses their affection and responds to my feelings, such as anger, grief, and love	29	8.0	112	30.8	223	61.3
Resolve	I5- I am satisfied with the time I spend with my family	42	11.5	129	35.4	193	53.0

Caption: I, item; n, total sample.

As for the “Almost never” option, item 5 (Decision) presented the highest percentage, with 11.5 %, followed by item 4 (Affection), with 8.0 %. The lowest value in this category was recorded in item 1 (Adaptation), with 3.8 %.

Regarding the categorization of the perception of FF, of the total sample (n=364), the majority (76.4 %) fell into the highly FF categorization and the smallest group into the family with severe dysfunction categorization (6.3 %). The average APGAR Index score was 7.82 ± 2.470 points, with a median of 9 points and mode of 10 points, with a minimum of 0 points and a maximum of 10 points.

The average of the General HL Index differed among users with different perception of FF (ANOVA: η^2^=0.019; p=0.031), with users who perceived their family as a “Highly functional family” obtaining the highest average than users who perceived their family as a “Family with severe dysfunction” (33.91>29.50). In the ANOVA test, a statistically significant effect was observed, with an eta squared η^2^ of 0.019, corresponding to a small effect. In other words, users who perceived their family as highly functional had higher HL. The statistical mean ranking of the HL categorization differed among users with different perception of FF (Kruskal-Wallis: p=0.003), with users who perceived their family as a “Highly functional family” obtaining a higher-ranking statistical mean than users who perceived their family as a “Family with severe dysfunction” (191.82>129.09). In the Kruskal-Wallis test, a statistically significant effect was observed, with an epsilon squared ε^2^ of 0.028, corresponding to a small effect. In other words, users who perceived their family as highly functional presented higher HL (Table 6).

**Table 6:** Results of statistical tests between general HLI and FF perception and between HL categorization and FF perception (n=364).

Variables	Af	Mean	df	Test value	p-Value	Eta/epsilon
General HLI^a^ family functionality						
Highly functioning family	278	33.91	363	ANOVA=3.508	0.031	η^2^=0.019
Mildly dysfunctional family	63	32.54				
Severely dysfunctional family	23	29.50				
HLI Cat^a^ family functionality						
Highly functioning family	278	191.82	2	KW=11.923	0.003	ε^2^=0.028
Mildly dysfunctional family	63	160.88				
Severely dysfunctional family	23	129.09				

Caption: Af, absolute frequency; df, degree of freedom; HLI Cat, health literacy index categories; HLI, health literacy index; p, significance level; KW, Kruskal-Wallis.

The coefficient and significance of the ordinal model were determined according to the *positive log-log* link function, expressed as **log[-log (1 - P(Y≤k))]**. In this expression, **Y** represents the ordinal dependent variable (HL), **k** corresponds to each of its categories (except the last one), and **P(Y≤k)** indicates the cumulative probability of the dependent variable taking on a category equal to or lower than **k**. The estimated coefficients for the model were

$$\alpha_{k}=(-1.792_{\text{family}\;\text{with}\;\text{severe}\;\text{dysfunction}}\;\;0.839_{\text{family}\;\text{with}\;\text{mild}\;\text{dysfunction}}+0.440_{\text{male}\;\text{sex}}+0.297_{\text{age}\;\text{group}\;24–33\;\text{years}}{\;\;1.468}_{\text{Basic}\;\text{Education}}\;\;0.587_{\text{secondary}\;\text{education}}{\;\;0.698}_{\text{single}\;\text{marital}\;\text{status}}\;\;0.731_{\text{married}/\text{common}‐\text{law}\;\text{status}}).$$

The reported statistics include G^2^, corresponding to the chi-square statistic of the model, and the pseudo-R^2^ coefficients: R^2^_MF_ (McFadden), R^2^_n_ (Nagelkerke), and R^2^_CS_ (Cox and Snell). The beta coefficients for the independent variables were also considered, namely b_FF_ for Family Functioning and b_(educational qualifications – Basic Education)_ for educational level.

The model was statistically significant (G^2^ (16)=33.646; p=0.06), although the effect size was small (R^2^_MF_=0.036; R^2^_N_=0.096; R^2^_CS_=0.088). According to the model, as the category of “severe dysfunction” decreases, the probability of observing higher HL classes increases (b_FF_=−1.792; p=0.008). Similarly, as the level of educational qualifications decreases (Basic Education), the probability of observing higher HL classes increases (b _educational qualifications Basic Education_=−1.468; p=0.030) (Table 7).

**Table 7:** Results of ordinal regression between the HLI categorization and the FF perception and sociodemographic categories.

Variables	Estimate	Wald	df	p-Value
**HLI cat^a^ family functionality**				

Highly functioning family	−1.792	7.137	1	**0.008**
Mildly dysfunctional family	−0.839	3.114	1	0.078
Severely dysfunctional family	0		0	

**HLI cat^a^ gender**				

Male	0.044	0.022	1	0.883
Female	0		0	

**HLI cat^a^ age group**				

≥24–33 years	0.297	0.818	1	**0.366**
≥34–44 years	0		0	

**HLI cat^a^ literary qualifications**				

Basic education	−1.468	4.719	1	**0.030**
Secondary school	−0.587	3.312	1	0.069
Higher education	0		0	

**HLI cat^a^ marital status**				

Single	−0.698	1.470	1	0.225
Married/common-law union	−0.731	1.980	1	0.159
Divorced/widowed	0		0	

Caption: df, degree of freedom; HL Cat, health literacy categories; p, significance level. All values with statistical differences are in bold.

## Discussion

The results of the present study revealed relevant patterns regarding HL levels and the perception of FF. A statistically significant relationship was identified between HL and FF, indicating that individuals with higher HL levels tended to report more favorable perceptions of FF.

The sample was predominantly composed of female participants aged between 34 and 44 years, with higher education and married or common-law union status. Although the proportion of users with non-limited HL was slightly higher, a very similar percentage of limited HL was observed. Most participants perceived their families as highly functional, reflecting a generally positive evaluation of FF.

Regarding HL levels, the sample presented similar proportions of problematic and sufficient General HL, with sufficient HL also predominating across the HC, DP, and HP dimensions.

Concerning the General level of HL, the results align with prior studies. Pedro et al. [23] reported similar proportions of problematic and sufficient HL (39.0 and 36.5 %). Consistent with this pattern, sufficient HL predominated in Arriaga et al. [7] (65 %) and Coman et al. [29] (59.2 %).

Comparable distributions were described by Pigazzini et al. [30] (7.9 % inadequate; 23.5 % problematic; 68.5 % sufficient) and Berens et al. [31] (8.18 % inadequate; 30.94 % problematic; 60.9 % sufficient). These findings highlight the predominance of sufficient HL, while confirming the persistent presence of limited HL across European populations.

In contrast, elevated proportions of problematic HL were observed in Lopes [32] (56.0 %) and Veladas [33] (45.0 %). Comparable distributions were reported by Guggiari et al. [34] (45.0 % problematic HL; 9.0 % inadequate HL). Such discrepancies may be partly attributable to differences in sample characteristics, particularly age profiles.

This interpretation is supported by broader European evidence, with Sørensen et al. [35] reporting limited HL levels ranging from 48.0 to 58.0 %, reinforcing the persistent prevalence of problematic HL.

Across the HC, DP, and HP dimensions, sufficient HL predominated. Arriaga et al. [7] reported comparable distributions (71.6 , 54.1, 54.6 %), while Lopes [32] observed elevated problematic HL (59.0 , 58.0, 45.0 %).

The HP dimension showed the most favorable distribution of sufficient HL, while the HC dimension presented the highest proportion of excellent HL, findings consistent with Veladas [33] (48.0 % sufficient HL in HP; 13.0 % excellent HL in HC). In contrast, Arriaga et al. [7] reported the highest sufficient HL in HC (71.6 %) and the highest excellent HL in DP (9.5 %). These variations may be partly explained by differences in sample characteristics.

Consistent with European evidence, distinct patterns across HL dimensions have been described, with stronger performance in HP and greater vulnerability in DP (Sørensen et al. [35]; Guggiari et al. [34]). The findings of the present study follow this established trend.

Regarding FF perception, a predominance of highly functional families was observed. Similar distributions were reported by Mota, Caramelo and Carvalho [36] (71.0 % highly functional; 10.0 % severely dysfunctional) and Teixeira [37] (71.1 % highly functional; 5.0 % severely dysfunctional), reinforcing the consistency of these findings.

Analysis of the Family APGAR items indicated that Item 1 (Adaptation) showed the highest proportion of “Almost always” responses, whereas Item 5 (Decision) presented the highest proportion of “Almost never” responses. Comparable patterns were reported by Fernandes and Mota, Caramelo and Carvalho [36], with high frequencies of “Almost always” responses for Item 1 (93.3 and 78.0 %) and lower proportions of “Almost never” responses for Item 5 (11.5 and 1.7 %).

In the present study, a positive relationship was identified between HL and FF, consistent with the findings of Niedorys-Karczmarczyk et al. [38], who also reported a significant association between these variables. While previous studies have examined the relationship between HL and FF, limited evidence exists using the Family APGAR scale. In this context, the present study contributes to expanding the understanding of the association between HL and family dynamics.

In conclusion, we can affirm that there is a relationship between the level of HL and the level of perception of FF, as well as the level of Educational Qualifications, in which the probability of observing higher HL classes increases when severe dysfunction decreases and when the level of Educational Qualifications also decreases. Therefore, users with severe dysfunction and greater family dysfunction and lower Educational Qualifications will have a lower HL level and will be groups that will need greater support and monitoring from the health team.

The understanding that a lower level of HL is associated with a less positive perception of family dynamics and lower Educational Qualifications allows health professionals to identify groups in greater vulnerability and direct more effective interventions. These data reinforce the need for educational and communication strategies adapted to the users’ literacy level, promoting not only individual empowerment, but also cohesion and support within the family unit, with positive impacts on health management and adherence to therapeutic plans.

This finding highlights the relevance of family-centered nursing interventions, emphasizing the potential contribution of the Family Nurse in promoting adaptive family dynamics.

The main limitations of this study include the use of a non-random sample, which limits generalizability, and the cross-sectional design, which precludes causal inference.

Overall, the findings reinforce the clinical relevance of HL and FF assessment, supporting the need for tailored educational and communication strategies that consider both individual and family contexts.

## References

[1] Arriaga M, Duarte S, Martins M, Paiva T (2021). Health Literacy Survey 2019 Portugal. Níveis de literacia em saúde.

[2] Brito MB, Barroca M, Esteves SL, Abrunheiro S (2023). Uma Evolução dos Conceitos da Literacia em Saúde. Manual de Literacia em Saúde: Princípios e Práticas. 1st ed. Pactor - Edições de Ciências Sociais.

[3] Direção-Geral da Saúde (2019). Plano de Ação para a Literacia em Saúde: (2019-2021). ..

[4] World Health Organization (2013). Health literacy: the solid facts.

[5] Vaz de Almeida C, Lopes C, Pinheiro D, Vital Brito D, Godinho LV, Subedi S (2023). Primeira revista de literacia em saúde da SPLS.

[6] Pelikan JM, Röthlin F, Ganahl K, Slonska Z, Doyle G, Fullam J (2020). Health literacy in Europe: comparative results of the European Health Literacy Survey (HLS-EU). Eur J Publ Health.

[7] Arriaga M, Francisco R, Nogueira P, Oliveira J, Silva C, Câmara G (2022). Health literacy in Portugal: results of the health literacy population survey project 2019-2021. Int J Environ Res Publ Health.

[8] Santos ESQdos (2021). Literacia em saúde na população do ensino superior da Universidade de Coimbra.

[9] Figueiredo MH (2012). Modelo Dinâmico de Avaliação e Intervenção Familiar.

[10] Castaño Castrillón JJ, Páez Cala ML (2019). Funcionalidad familiar y tendencias adictivas a internet y a sustancias psicoactivas en estudiantes universitarios.

[11] Ferreira Y, Santos L, Brito T, Rezende F, Neto L, Osório N (2019). Funcionalidade Familiar e sua relação com fatores biopsicossociais. Humanidades e Inovação.

[12] Yeom HE, Park DS (2024). Mediating and moderating effects of uncertainty on the relationship between family function, self-care, and depression among blood cancer survivors. Behav Sci.

[13] Santos M, Vilaça M, Portugal A, Relvas AP (2021). Funcionamento familiar: Revisão de estudos empíricos sobre medidas de avaliação (FAD, FACES-IV e SCORE-15). Rev Iberoam Diagn Eval.

[14] Smilkstein G (1978). The family APGAR: a proposal for a family function test and its use by physicians. J Fam Pract.

[15] (2014). Decreto-Lei no. 118/2014, de 5 de agosto. Diário da República, 1a série.

[16] Ferreira YCF, Santos LF, Brito TRP, Rezende FAC, Silva Neto LS, Osório NB (2019). Funcionalidade familiar e sua relação com fatores biopsicossociais. Revista Humanidades & Inovação.

[17] Vilelas J (2022). Investigação: o Processo de Construção do Conhecimento.

[18] (2017). Decreto-Lei no. 73/2017, de 21 de junho. Diário da República, 1.a série.

[19] (1979). Lei no. 56/79, de 15 de setembro. Diário da República, 1.a série.

[20] Instituto Nacional de Estatística (INE) (2024). Inquérito à Utilização de Tecnologias da Informação e da Comunicação pelas Famílias 2024.

[21] Autoridade Nacional de Comunicações (ANACOM) (2024). Competências digitais da população e das empresas 2024.

[22] Araldi AA (2004). Processo de amostragem tamanho da amostra.

[23] Pedro AR, Raposo B, Luís L, Amaral O, Escoval A, Simões Dias S (2023). Portuguese version of the HLS-EU-Q6 and HLS-EU-Q16 questionnaire: psychometric properties. Int J Environ Res Publ Health.

[24] Smilkstein G, Ashworth G, Montano D (1982). Validity and reliability of the family APGAR as a test of family function. J Fam Pract.

[25] Agostinho M, Rebelo L (1988). Família: do conceito aos meios de comunicação. Revista Portuguesa de Saúde Pública.

[26] Sørensen K, Van den Broucke S, Fullam J, Doyle G, Pelikan J, Slonska Z (2012). Health literacy and public health: a systematic review and integration of definitions and models. BMC Public Health.

[27] Pedro AR, Amaral O, Escoval A (2016). Literacia em saúde, dos dados à ação: tradução, validação e aplicação do European Health Literacy Survey em Portugal. Revista Portuguesa de Saúde Pública.

[28] Marôco J (2020). Análise estatística com o SPSS statistics.

[29] Coman MA, Forray AI, Van den Broucke S, Cărăreş RM (2022). Measuring health literacy in Romania: validation of the HLS-EU-Q16 survey questionnaire. Int J Publ Health.

[30] Pigazzini S, Vogt DR, Wieser S, Wang S (2024). Inadequate health literacy and higher healthcare utilisation among older adults in Switzerland: cross-sectional evidence from a population-based study. Swiss Med Wkly.

[31] Berens EM, Vogt D, Messer M, Hurrelmann K, Schaeffer D (2023). Associations of health literacy with socioeconomic position, health behaviour and health status: a cross-sectional survey of 9007 adults from the German National Health Interview and Examination Survey. BMC Public Health.

[32] Lopes MFB (2022). Literacia em saúde dos utentes de um centro de saúde da região Norte.

[33] Veladas FMV (2022). Literacia em saúde: aplicação do European Health Literacy Survey e do Oral Health Adults Questionnaire em duas populações de diferentes faixas etárias.

[34] Guggiari E, Jaks R, Berger FMP, Nicca D, De Gani SM (2021). Health literacy in the canton of Zurich: first results of a representative study. Int J Environ Res Publ Health.

[35] Sørensen K, Pelikan JM, Röthlin F, Ganahl K, Slonska Z, Doyle G (2023). Health literacy and public health: results of the European Health Literacy Population Survey (HLS19) in nine European countries. BMC Public Health.

[36] Mota M, Caramelo A, Carvalho A (2023). Perceção do idoso sobre a funcionalidade familiar. Revista de Investigação & Inovação em Saúde.

[37] Teixeira RMS (2020). Gestão do regime terapêutico de utentes diabéticos: funcionalidade familiar e intervenção do enfermeiro de família [dissertation]. Universidade de Trás-os-Montes e Alto. Douro.

[38] Niedorys-Karczmarczyk B, Nowicki GJ, Chrzan-Rodak A, Nowak-Starz G, Ślusarska BJ (2024). Health literacy levels and their sociodemographic, family, and health predictors among primary health care patients in urban and rural areas: a cross-sectional study. Med Stud/Studia Medyczne.

